# Cylindrospermopsin: A Decade of Progress on Bioaccumulation Research

**DOI:** 10.3390/md8030542

**Published:** 2010-03-09

**Authors:** Susan Kinnear

**Affiliations:** Centre for Environmental Management, CQUniversity Australia, Building 7, Bruce Highway, North Rockhampton, Queensland, 4702, Australia; E-Mail: s.kinnear@cqu.edu.au; Tel.: +61-(0)7 4930 3996; Fax: +61-(0)7 4930 9156

**Keywords:** Cylindrospermopsis raciborskii, ecotoxicity, microcystin, deoxy-cylindrospermopsin, nostocales, freshwater

## Abstract

Cylindrospermopsin (CYN) is rapidly being recognised as one of the most globally important of the freshwater algal toxins. The ever-expanding distribution of CYN producers into temperate zones is heightening concern that this toxin will represent serious human, as well as environmental, health risks across many countries. Since 1999, a number of studies have demonstrated the ability for CYN to bioaccumulate in freshwater organisms. This paper synthesizes the most current information on CYN accumulation, including notes on the global distribution of CYN producers, and a précis of CYN’s ecological and human effects. Studies on the bioaccumulation of CYN are systematically reviewed, together with an analysis of patterns of accumulation. A discussion on the factors influencing bioaccumulation rates and potential is also provided, along with notes on detection, monitoring and risk assessments. Finally, key gaps in the existing research are identified for future study.

## 1. Introduction

Cyanoprokaryotes (cyanobacteria, blue-green algae) are non-nucleated organisms which lack membrane-bound organelles and sexual reproduction [1], but are characterized by their ability to synthesize chlorophyll a [2]. The cyanoprokaryotes are a diverse and adaptive taxon, occupying a broad range of ordinary aquatic environments as well as extreme habitats such as Antarctic ice shelves and volcanoes. However, they are most commonly of interest as a component of the phytoplankton populations of freshwater, estuarine and marine environments. Of particular economic, human and environmental importance are those species known to form blooms, especially where these are associated with the production of toxins.

Toxic blue-green algal blooms from both marine and freshwaters are now readily acknowledged as a serious human health risk [3]. The potential for cyanotoxins to cause serious health effects has elevated them into the consciousness of water managers worldwide. Health risks are posed by swimming or partaking in recreational activities in water bodies; by drinking unsuitably treated water or consuming toxin-laden tissues; *via* possible ingestion in dietary supplements; or a combination of these [3–6]. In addition to the human health threat, cyanotoxins are also emerging as an environmental health concern. In this context, toxins exert acute and chronic lethal and sublethal effects on a range of terrestrial and aquatic plants and animals [7–11].

Bioaccumulation of toxins is a separate issue to environmental toxicity, though there are likely to be direct relationships between toxin accumulation and the nature and strength of toxic effects. Bioaccumulation occurs where tissue-based concentrations exceed those available in the environment: the latter may include algal toxins available through drinking, dietary, and/or direct contact routes. Uptake can potentially occur *via* plant surfaces or dermal exposure (skin or gills), oral consumption of cells or contaminated tissues, and/or (accidental) drinking of suspended particles and aqueous concentrations. Toxin adsorption may also occur: this allows toxin to become associated with the tissues of aquatic biota, though not necessarily being truly intracellular. Biomagnification, where toxin concentrations are increased through successive trophic level interactions, may also be possible.

Many aquatic organisms (phytoplankton, zooplankton, plants and animals) experience direct contact with aqueous (lysed or leaked) toxins in the water column during an algal bloom; many also are vulnerable to ingestion of toxin-laden cells *via* algal grazing or accidental drinking. Thus, the uptake potential for any of the cyanotoxins is considerable. The potential for algal toxins to bioaccumulate has received some attention in the last decade, particularly so with respect to accumulation in fish, crustacean or other seafood species with recreational or commercial importance [12]. A review of bioaccumulation of cyanotoxins and their effects on aquatic organisms can also be found in Filho (this volume). However, many of these have concentrated on the hepatotoxin microcystin. Far fewer studies have been conducted on cytotoxin, cylindrospermopsin (CYN), despite the fact that the predominantly extracellular availability of this toxin makes it particularly likely to be taken up by a variety of aquatic organisms. A lack of readily available CYN material, and/or *C. raciborskii* lyophilates, is one of the predominant reasons for this anomaly.

## 2. Cylindrospermopsin

### 2.1. Properties

Cylindrospermopsin (CYN) is a tricyclic alkaloid cytotoxin first isolated and identified in 1992 [13]. Structural variants include 7-epi-CYN and 7-deoxy-CYN [14,15]; the toxin also exists in the deoxygenated form, deoxy-CYN [16]. The toxin molecule is a sulfated guanidinium zwitterion and is stable in varying heat, light and pH conditions [17]. It is also highly water-soluble, and has a relatively low molecular weight of 415 Daltons [18,19].

### 2.2. Distribution and detection

**Figure 1:**
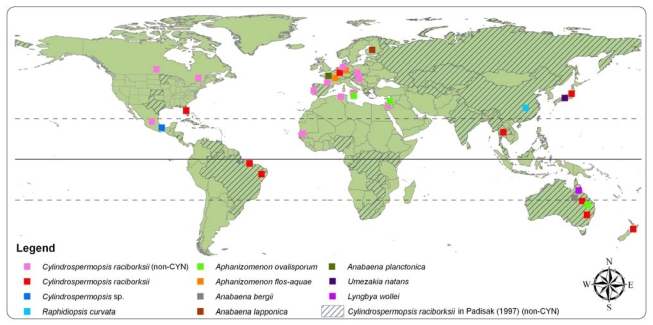
Global distribution of algal blooms known to contain CYN or a CYN-analog. Note: ‘non-toxic’ denotes a bloom from which toxicity was not confirmed or not studied; figures are not exhaustive as some records from central and eastern United States not shown. Collated from [19,20,23–59].

Cylindrospermopsin production has been recorded from several Nostocalean species as well as recently from one Oscillatoriale [20]. The principal species for CYN production is *Cylindrospermopsis raciborskii*, the namesake of the toxin. *C. raciborskii* can occupy a diverse range of environments including intensively-flushed lotic bodies and newly built reservoirs [21]. The distribution of *C. raciborskii* was reviewed by Padisák [21], who catalogued blooms occurring in tropical and subtropical nations as well as those expanding into temperate climes. However, whether CYN co-occurred at most of these sites was not validated. New reports have also been made of other CYN, deoxy-CYN and epi-CYN producers including *Raphidiopsis*, *Aphanizomenon*, *Anabaena*, *Umezakia* and *Lyngbya; Aph. gracile* has recently been flagged as yet another CYN producer [22]. The toxin is thus now reported from Asia, Africa, North and South America, central, southern and northern Europe, and Australia/New Zealand–every continent except the Antarctic (Figure 1). The toxin is now approaching an almost cosmopolitan distribution pattern and CYN producers are recorded from habitats including lakes, reservoirs, rivers, ponds and dams. Nevertheless, it is expected that many locations in which CYN is present will remain undetected, as some producer organisms rarely form visible blooms or surface, even during intense blooms (e.g., *C. raciborskii* and benthic *L. wollei*) [23].

According to Kling [23], improvements in water quality monitoring is a key contributor to the number of new locations from which *C. raciborskii* has been recorded. However, it is also likely that the organism is expanding into suitable habitats made newly available by a combination of climate change, increased eutrophication and the species’ own adaptability [23]. Padisák [21] noted that the ability of *C. raciborskii* to travel long river courses, to survive swampy or slightly saline conditions, and to produce resistant akinetes has contributed to expansion of this species on a global scale. Global climate change has also been examined as a trigger for the increasingly widespread distribution, frequency and duration of *C. raciborskii* blooms, especially into the sub-tropical and temperate regions [34,44,60–62]. Here, the effects of climate change are much more insidious than simply an increased upper limit of surface temperatures. For example, winter warming coupled with increased evapotransporation has led to reduced water volumes in the Mediterranean, thus encouraging a stable water column conducive to *C. raciborskii* blooms [35]. Furthermore, changes in, and intensification of, land use activities within various catchment areas worldwide is also associated with an increased incidence of blue-green domination [35]. In future, it is likely that the combined effect of these influences will mean an ever-increasing area from which CYN will be recorded.

Harada *et al.* [63] developed the first screening method for CYN using reverse phase high performance liquid chromatography (HPLC) coupled with photo diode array detection. CYN has an easily identifiable peak and maximum UV absorbance at 262nm [13,64]. The use of HLPC/mass spectrometry with electrospray results in a detection limit of 200 μg L^−1^ [65], but when combined with tandem mass spectrometry, a 1 ug L^−1^ limit can be achieved [66,67]. Whilst the use of HPLC with photo diode array is a less expensive alternative to MS/MS, it may not adequately detect trace quantities of CYN [68]. Reliable PCR systems have been demonstrated [54] and immunochemical techniques such as ELISA kits are also newly available for CYN [69]. These approaches have made the detection of CYN in water a faster and more discerning process.

### 2.3. Studies on mechanism of toxicity

The effects of CYN have been studied in mammalian species, or their target organs and cells [33,56,70–75]. More recent studies of CYN have expanded toxicity models to show effects in invertebrates, zooplankton, phytoplankton, bacteria and protozoans [76–79]. There is considerable variability amongst the toxicity of CYN between different animal models [80] and even between different individuals of the same species [71]. Plant studies have been limited to only three reports on tobacco, duckweed and Hydrilla species [81–84].

An excellent summary of research progress with respect to the mechanisms of toxicity of CYN is provided in Humpage [85], so a similarly detailed description is not attempted here. Generally, however, toxin exposure is characterized by delayed toxicity involving multiple organ systems, principally the liver and kidney. Toxicity is mediated by inhibition of protein synthesis, as well as genotoxicity by DNA fragmentation [85,86]. It is clear that the metabolic activation of CYN is linked with higher toxicity, though the precise pathway for this remains unclear [87]. Interestingly, it is this nature of CYN (*i.e.*, where toxin metabolism must occur before full toxicity is imparted) that also offers some protection to exposed species. For example, plants and animals lacking an advanced toxin metabolism system (e.g., liver or hepatopancreas) typically feature reduced toxicity, as do the early developmental stages of mammalian species [74]. Notably, to date, CYN remains unique amongst the algal toxins in causing toxicity *in utero*, being linked with premature births, reduced size and increased mortality in mice pups [74]. It has also been recognized that CYN has some potential for endocrine disruption: one study showed the toxin could alter the progesterone:estrogen ratio in women, although this result must be interpreted with caution given the variability amongst the tested individuals [88].

### 2.4. Human health effects

Along with other algal toxins, the human health effects of CYN are summarized in a devoted volume by Hudnell [89] as well as several earlier accounts [10,24]. Since these reports, the majority of recent advances in CYN research have been directed at improving the understanding and estimation of human health risks, as well as the ability to assess and forecast situations where CYN may present human health threats. However, whilst these aspects of CYN toxicity are of obvious concern, they are not the focus of this review.

### 2.5. Ecological effects

Comparatively speaking, ecological studies have lagged far behind those for human health and risk assessments: this is true of every cyanotoxin. However, unlike other toxins such as microcystin (which is largely hepatotoxic), CYN is widely cytotoxic, as the primary mechanism of CYN is protein synthesis inhibition. This means that CYN has the potential to impact a wide variety of aquatic and semi-aquatic plant and animal species. Furthermore, because bioaccumulation and trophic transfer is possible with CYN, toxicity may also be exerted on first, second and higher-order consumers in aquatic food webs. This could expand CYN’s environmental damage to include largely terrestrial organisms. Thus, the need to more closely study the effects that CYN has a wide range of organisms is clearly evident.

Seifert [78] demonstrated that significant adverse effects were rarely recorded for CYN exposure concentrations below 100 μg L^−1^ of pure toxin. This was true for a range of species including a floating macrophyte, green algae, and a range of aquatic invertebrates from various trophic levels. On the other hand, exposure to *C. raciborskii* extracts resulted in greater sub-lethal toxicities [78]. This suggests that cell extracts–and hence, field populations of CYN-producing blooms–are likely to contain one or more bioactive compounds other than CYN, so increasing the risk of toxic effects. This situation has also been documented in a number of other studies [16,64] and was discussed in Kinnear *et al.* [90]. In the future, performing work with antibodies may help to shed light on whether these unidentified compounds are CYN analogs [87].

Detailed notes on the factors moderating exposure to, and toxicity of CYN produced by *C. raciborskii* were made in Kinnear *et al.* [90]: these included the occurrence and cell concentrations of a bloom; the dynamics of toxin production including concentration and bioavailability; toxin uptake pathways; which organisms are likely to be exposed; individual susceptibilities; the potential for bioaccumulation; and whether synergies would exist because of other toxins being present, or due to deteriorated water quality during a bloom.

## 3. Bioaccumulation of CYN

### 3.1. Existing evidence

A decade has passed since the first report was made of CYN bioaccumulation by Saker & Eaglesham [91]. Since then, the total number of published studies on CYN accumulation has risen to more than ten. Within these, bioaccumulation of CYN has been shown from a range of invertebrate and vertebrate organisms including *Melanoides tuberculata* and Tegogolos snails; *Anodonta*, *Alathyria* and *Corbiculina* mussels; *Cherax* crayfish, *Melanotaenia* rainbowfish and *Bufo marinus* tadpoles [48,91–95]. In plants, bioconcentration of CYN (*i.e.*, accumulation resulting from the uptake of aqueous toxin only) has been studied in two types of duckweed (*Lemna* and *Spirodela*) as well as Hydrilla, though only *Lemna* actually concentrated the toxin [78,83,96].

Saker and Eaglesham [91] first studied bioaccumulation in crayfish *Cherax quadricarinatus* and rainbow fish *Melanotaenia eachamensis.* In the crayfish, toxin was detected from both the muscle (900 μg kg^−1^ freeze-dried tissue) and hepatopancreatic tissues (4,300 μg kg^−1^ freeze-dried tissue) after specimens were collected from an aquaculture pond containing 589 μg L^−1^ CYN. Toxin was also recovered from the visceral tissue of rainbow fish *Melanotaenia eachamensis* at a maximum of 1,200 μg kg^−1^ freeze-dried tissue). Laboratory trials with the crayfish demonstrated bioaccumulation could occur within fourteen days of toxin exposure, with hepatopancreatic and abdominal tissues containing over 1,000 and 200 μg toxin kg^−1^ freeze dried tissue, respectively [91].

Norris *et al.* [97] showed CYN accumulated into the liver and kidney of mice within six hours of dosing *via* IP injection. The accumulation rate progressively decreased over five to seven days, after a single dosing event. The authors also demonstrated that up to 73% of toxin was excreted in urine and/or faeces within twelve hours. However, whole-of-organism bioaccumulation factors were not reported in the study.

Anderson *et al.* [95] demonstrated CYN accumulation of 130–560 μg CYN kg^−1^ fresh from *Alathyria pertexta pertexta* following environmental exposure to reservoir water containing <0.8 μg L^−1^ toxin.

Nogueira *et al.* [98] reported CYN from the tissues of the cladoceran Daphnia magna following exposure to *C. raciborskii.* However, bioaccumulation was not evident, as bioaccumulation factors (BAFs) were below one (0.71 and 0.46; for 24 h and 48 h exposures, respectively).

Saker *et al.* [94] exposed the swan mussel, *Anodonta cygnea*, to total CYN values ranging from 14–90 μg L^−1^ over sixteen days. This resulted in maximum dry weight tissue accumulations of 61,500 μg kg^−1^ in the haemolymph; 5,900 μg kg^−1^ in the viscera; and 2,900 μg g^−1^ for whole-body tissues. Toxin was also detected in tissues of the mantle, foot and gonad. The relative distribution of CYN in the tissues changed over the trial period, although bioaccumulation generally occurred within two days’ exposure [94]. The authors also studied the mussels during a fourteen-day depuration period, after which time almost 50% of the toxin remained in the tissues. Depuration was found to be bi-phasic and marked by small increases in tissue toxin concentrations: these were thought to indicate mobilisation of tissue-bound CYN [94].

In 2007, Kinnear *et al.* [82] published the first study examining CYN accumulation into plant tissues. Duckweed was exposed to CYN concentrations in the range 0–120 μg L^−1^. Whilst nanogram quantities of toxin were recovered from the plant tissues, this was not sufficiently high to indicate that bioconcentration has occurred after either six or twelve day’s exposure. Rather, the detected toxin was considered likely to result from toxin adsorption to the plant cell walls [82]. Later work with *Hydrilla verticillata* also showed that bioconcentration did not occur when the submerged plants were exposed to CYN, even with exposure concentrations up to 400 μg L^−1^ [96]. For example, a maximum of 176 ng g^−1^ CYN was detected from the plant tissues, representing a bioconcentration factor (BCF) of only 0.045.

Doctoral studies conducted by Seifert [78] established that bioaccumulation of both CYN and deoxy-CYN occurs in field populations of eel-tailed catfish (*Tandanus tandanus*), but not in other species including golden perch (*Macquaria ambigua*), silver perch (*Bidyanus bidyanus*) *or* Australian bass *(M. novemaculeata*). Seifert re-confirmed that bioaccumulation occurs in *Cherax* crayfish, as had already been reported by Saker & Eaglesham [91]. He also reported toxin from *Corbiculina australis* mussels, where, interestingly, BAFs for deoxy-CYN were an entire order of magnitude higher than those for CYN (e.g., 810–1,600 compared with 13–23 for muscle and viscera, respectively). Each of these species was collected opportunistically from Australian field sites experiencing known CYN-producing blooms.

Seifert (2007) also recorded CYN bioconcentration from duckweed (*Lemna punctata*), but only where pure exposure concentrations reached 570 μg L^−1^, but even then, this was barely so with a maximum BCF of 1.08 [78]. However, where cell extracts were used rather than purified toxin, substantially higher BCFs were recorded, at up to 86.67.

In White *et al.* [92], laboratory studies conducted over seven and fourteen days with *Melanoides tuberculata* showed that snails were certainly capable of accumulating toxin. With exposure concentrations ranging from 91–406 μgL^−1^, the maximum BAF was 124. However, the availability of intracellular toxin was critical in affecting the levels of CYN recovered from the snail tissues: accumulation was hardly recorded following exposure to cell extracts containing aqueous toxin; whereas much higher quantities were recorded during exposure to live cultures of *C. raciborskii* [92]. The amount of accumulated toxin was also highly variable between different snails. Deoxy-CYN was also recorded from the snail tissues, with a maximum BAF of almost 250. This occurred despite the exposure concentrations being much lower than for CYN (e.g., 3–12 μg L^−1^) [92]. Lastly, small quantities of toxin were also recovered from the snails’ shells, although this was not likely to be related to bioaccumulative activity.

In White *et al.* [93], seven-day laboratory studies were conducted with tadpoles of the cane toad, *Bufo marinus*. After being exposed to whole cell extracts containing up to 400 μg L^−1^ aqueous CYN, toxin was recovered from the tadpole tissues, but not in sufficient quantities to demonstrate bioconcentration [93]. In contrast, tadpoles accumulated up to 895 μg CYN kg^−1^ fresh weight when they were exposed to live *C. raciborskii* cultures containing 232 μg L^−1^ CYN, with BAFs of up to 19.27. However, the rates were highly variable, both between different trials and amongst different treatments. Deoxy-CYN concentrations were not reported from the tadpole tissues during the study, but this probably reflected the typically low exposure concentration (maximum of 7 μg L^−1^).

The most recent study of CYN bioaccumulation has been conducted by Berry & Lind [48], which reported CYN accumulation from Tegogolo snails (*Pomecea patula catemacensis*) from field environments. Here, tissue toxins were detected at 3.35 ± 1.90 ng g^−1^ using ELISA techniques. This represented at bioaccumulation factor of 157. Importantly, these values were recorded in conjunction with environmental concentrations of just 20 ng L^−1^ CYN, thus demonstrating that bioaccumulation can occur even at exceptionally low field concentrations

### 3.2. Patterns of toxin uptake and deposition

The pathway of CYN uptake is poorly understood; most work on this aspect of the toxin has been carried out with respect to toxicity, rather than accumulation potential. Past work by Runnegar *et al.* [99] showed that whilst the sulfate group on the CYN molecule is not required for cell entry, the hydrophilic nature of the CYN makes the molecule unlikely to cross cell walls. Nevertheless, the small molecular weight of CYN makes passive diffusion a real possibility [100]; recent work has resulted in diffusion across epithelial cells now being considered an important pathway for CYN uptake [101]. Damage to the gut lining of organisms ingesting CYN has also been demonstrated in several studies [71,73,77]. This is important in terms of uptake pathways, since it may accelerate CYN absorption, with broken digestive epithelia offering a greater surface area over which toxin uptake can occur.

In animal models, the CYN uptake system is dependent on bile acid transporters during the initial stages, though Chong *et al.* [100] believed a secondary system was likely, since bile acid inhibition gives protection against CYN toxicity for 72 h only. Seifert [78] commented that uptake of CYN appears greater when the toxin is made available in the presence of other cellular compounds (e.g., cultured material compared with purified toxin). This is of obvious importance for future studies using purified toxin, given that such work could be compromised from the perspective of environmental relevance.

The fact that CYN accumulates preferentially into particular tissues was first demonstrated by Saker & Eaglesham [91], who reported the hepatopancreas tissues of crayfish to contain five times greater toxin concentration than the muscles. Seifert [78] also reported CYN and deoxy-CYN concentrations of the muscles to be twice that of those in the viscera of *Corbiculina* mussels. This indicates CYN’s likely affinity for blood or lymph, in contrast to other toxins such as microcystin and nodularin, which concentrate into the hepatopancreas/viscera [102]. Furthermore, as CYN is a highly water soluble molecule, bioaccumulation of toxin appears limited to the gut or to the liver due to active transport to the hepatocytes [48].

A high level of variability exists in CYN accumulation by different aquatic animals. Kinnear *et al.* [90] has already noted an emerging pattern whereby lower-level organisms accumulate greater concentrations of CYN toxin than do other, more biologically complex, animals. For instance, current evidence suggests the general order of bioaccumulation capacity being gastropods > bivalves > crustaceans > amphibians > fish. Curiously, the reverse relationship appears to be true for the susceptibility of organisms to CYN toxicity [90]; indeed, Smith *et al.* [127] noted that grazer species appear to be the most tolerant. This suggests that the toxic effects imparted during CYN exposure may have some bearing on the ability of organisms to accumulate the toxin. For example, it is conceivable that normal cell depuration and detoxification processes may be disrupted during or following exposure, therefore allowing toxin residues or metabolites to accumulate more easily in susceptible organisms. On the other hand, it could also be expected that species that are highly susceptible to CYN toxicity may reduce their grazing rates or simply die, thus minimizing accumulation. This could be true of fish and other aquatic vertabrates, since animals with highly advanced toxin-metabolism systems are at greater risk of secondary CYN toxicity [19,103]. Meanwhile, lower organisms such as aquatic snails can accumulate high levels of CYN without lethal effect [76].

As well as inter-specific differences, there is also considerable variability in the toxin concentrations accumulated into the tissues of conspecifics: this is true for *Bufo* tadpoles and *Melanoides* snails exposed to cultures of toxic *C. raciborskii* [92,93]. Again, this may reflect variation in the toxic effects of exposure; it may also reflect individual grazing rates (and thus toxin uptake) or changes in the proportion of toxin present in the intracellular form.

In plants, it is toxin adsorption, rather than true accumulation, which has been reported in almost all studies with CYN. Work to date has given no clues as to the possible uptake system(s) in primary producers. However, the characteristic nature of CYN to cause root stimulation in low-level doses [83,104] suggests that toxin transport to the roots may be possible, since roots buried in sediment are unlikely to come into contact with CYN suspended in the water column. This means that bioaccumulation studies must be conducted carefully. For example, toxin recovery must attempted for all plant parts of emergent macrophytes, regardless of their level of direct contact with water containing CYN.

The threat of CYN biomagnification was briefly discussed by Berry & Lind [48]: the authors noted that, given CYN is a largely water-soluble molecule, biomagnification was not considered likely. However, the quite separate issue of trophic transfer, where toxin is able to move throughout the food web, whilst not actually being accumulated, has already been shown for CYN [48]. This corroborates earlier work done by Seifert [78], which suggested that the toxin accumulated in catfish was actually sourced from freshwater bivalves. A focus on CYN biomagnification is thus particularly important for the future, especially given where toxin may be deposited in the flesh portion of animals consumed by humans.

### 3.3. Factors influencing bioaccumulation rates and potential

The global expansion of CYN through climate change, increasing eutrophication and the creation of more storage impoundments will create an increased prevalence of CYN producers. Ultimately, this will lead to a greater variety of plants and animals being exposed to the toxin. In turn, this may result in an increased incidence of bioaccumulation. In terms of at-risk locations, one study has shown that the toxin loading of *C. raciborskii* is higher in tropical compared with sub-tropical environments [60]. On the other hand, other studies have shown that toxin production is optimized in cooler waters [24]. Thus, there can be no easily-reached conclusion that temperate environments would represent a greater accumulation risk than elsewhere tropically. Moreover, linkages between temperature and toxicity will also affect the seasonality of bioaccumulation risk. For example, lengthening of bloom periods, and their incursion into the winter months (where toxin production may be much higher), will mean that different developmental stages of animals and plants will also be under threat.

It is also important to note that the presence of, or contact with, an active bloom is not necessarily a requirement for toxin accumulation. Firstly, Kinnear *et al.* [90] has considered the possibility that specialised cells, such as akinetes and heterocytes, may represent a greater or lesser role in mediating toxin accumulation, than do normal vegetative cells. Since akinetes are produced in large numbers at the end of a bloom and may settle into the sediments [105], filtering organisms could remain at risk of ingesting them well after the bloom has concluded.

Secondly, new Australian research has shown that very high CYN and deoxy-CYN concentrations can be recorded from the hypolimnion–with this toxin being both spatially and temporally distant from the producer cells [106]. This means that the risk period for bioaccumulation may be extended by weeks or even months, particularly if microbial and/or photocatalytic degradation is limited by the dark, anoxic environment. For example, copper sulphating of *A. ovalisporum* and *C. raciborskii* blooms results in slow loss of toxin over one to several months, probably because that treatment also slows microbial activity [50,107]. On the other hand, concentration of toxin into deep waters may also reduce bioaccumulation risk, given that few animals and plants would inhabit these dark anoxic waters. In addition, yet another study has shown that CYN degradation actually occurs in the sediments, not in a water body [108].

Thirdly, the possibility that crops irrigated by CYN-contaminated water must be considered as a further mechanism whereby bioaccumulation may occur. Reports of algal toxicity and toxin retention occurring under these conditions are already available for microcystin [109–111], though not for CYN.

The importance of the relative abundance of intracellular (cell-bound) and extracellular (aqueous or non-cell-bound) toxin in affecting bioaccumulation was discussed in Kinnear *et al.* [90], in the context of this ratio also moderating environmental toxicity. The discussion summarized earlier findings of studies with snails and tadpoles, which showed that exposure to high levels of intracellular toxin (e.g., through ingestion of toxin laden cells) can result in far greater accumulation rates and toxicity than when compared with aqueous toxin, or cellular extracts [92,93]. This is very important for toxic blooms of *C. raciborskii*, where it is not unusual for between 70 to 98% of CYN to be dissolved in the water column [65]. Extracellular CYN also accounts for a considerable amount of total CYN in laboratory cultures of *C. raciborskii*, especially those in the postexponential growth phase [24,129]). A new study by Orr *et al.* [112] is notable in being able to describe linkages between the genetic composition of field populations on *C. raciborskii* and intracellular CYN quotas: this could be useful in terms of genetic tests being capable of identifying blooms having particular CYN bioaccumulation risk. That study also shed further light on the relationship between *C. raciborskii* toxin cell quotas and cell concentrations; again, both of these are critical in influencing bioaccumulation. Unfortunately, neither laboratory nor field studies have yet been conducted on the split between intracellular and extracellular CYN production for any other of species known to be CYN-producers.

Other important dynamics in a bloom would include the toxin quota per cell, since higher cell loading would lead to greater toxin uptake (and thus bioaccumulative potential) in grazing and filter feeding animals. However, this would only be the case if the palatability of the cells remained unchanged and there were no other chemical cues by which grazing species could preferentially select against highly toxic cells. Indeed, grazing pressure itself may affect toxin quotas, although information on this is scant [92,113]. The positioning of algal cells is also likely to be important in affecting toxin exposure and uptake rates.

Lastly, many of the factors that govern the overall toxicity of CYN are also likely influencers on bioaccumulation. For example, in many aquatic and semi-aquatic organisms, a combination of transdermal uptake and accidental drinking of aqueous toxin, in conjunction with grazing on toxin-laden cells, is likely contribute to CYN entry into the tissues. These kinds of dynamics have already been recorded for other toxins [114,115]. The natural laws of size and surface area to volume ratios will thus apply with respect to uptake from direct (dermal) contact, so placing smaller animals at increased risk of transdermal toxin uptake. Already, it is known that microcystin toxin uptake rates are higher in moss compared with other aquatic macrophytes, because of the larger surface area to volume ratio coupled with the lack of protective cuticle [116]. Smaller organisms, including juveniles, may also have increased susceptibility to bioaccumulation because of limited mobility [117]. CYN accumulation is also likely to be affected by metabolic rates, as these affect consumption rates [118]. Differences in CYN accumulation rates of different organisms will also reflect the fundamental differences in uptake mechanisms and rates; different toxin transport mechanisms in plant and animals; and the differences in the ability to depurate toxin.

### 3.4. Field monitoring of bioaccumulation

The development of an early warning system for possible CYN bioaccumulation is important from the perspectives of both environmental and human health toxicity [119]. Molecular detection methods and genetic studies showing toxin-producing capability now lead contemporary research in CYN monitoring. For example, a large majority of algal toxin studies are now concentrating on the genetic composition of various species (e.g., isolating CYN synthase clusters), to determine their toxin producing capability (e.g., [31]). Cell culture-based toxicity tests are also being developed; these can be useful in detecting a range of toxins from fresh and marine waters [120]. Detection of CYN in water is also being optimized through new studies of more rapid and reliable testing techniques [121–123]. These will no doubt help to determine when bioaccumulation is likely to occur. Use of sentinel organisms, such as snails, to help monitor CYN in aquatic environments has also been suggested [48].

However, an entirely different problem is the proper quantification of CYN once it has become deposited in plant or animal tissues. This is an obvious necessity when determining the appropriateness of allowing for human consumption and in further studies on bioaccumulation. For example, CYN may bind easily to tissues because of its structural features [10]; similar problems are experienced with another algal toxin, microcystin [124]. In this situation, serious underestimation of the accumulative capability of CYN may result. However, toxin binding following CYN uptake has been hardly studied. Early indications reported in Froscio *et al.* [72] were that CYN was taken up into hepatocyte cell lines before becoming trapped.

Another concern is that new work with microcystin has shown that boiling of carp tissues results in generally three-fold higher values of microcystin recovery from the tissues, as well as causing the toxin to leach into the boiled water [125]. Like microcystin, CYN is also very stable in heat, thus, there is a very concerning possibility that CYN binding in fish tissues could be reversible, and that this ‘reversibility’ could be enhanced by cooking. Furthermore, CYN is more toxic once metabolically activated, [103] and the toxicity of different analogs (such as CYN compared with deoxy-CYN) is quite different [126]. Thus, it is conceivable that CYN ingested, accumulated, and then altered and made available *via* the cooking process may represent quite different, and possibly additional, health risks.

### 3.5. Risk assessments and bioaccumulation

Cylindrospermopsin is now one of the most keenly researched amongst the toxins produced by blue-greens: a Scopus database search for ‘cylindrospermopsin’ papers published between 2007–2010 alone returned some one hundred articles. A solid body of research is thus now available to underpin CYN risk assessments, but again, these are squarely focussed on human health risks. For example, national CYN guideline values and World Health Organisation limits for human health are variously available; tolerable daily intakes for adults, children and infants as well as livestock were presented in Duy *et al.* [10]. However, as more information about the genotoxic and carcinogenic effects of CYN comes to light, these trigger levels continue to be revised. New and more complete information on bioaccumulation, and the accompanying risk of ingesting toxin in seafood, should also warrant changes in such values. The need to include both CYN and deoxy-CYN in toxicity assessments and guidelines has been raised by Orr *et al.* [112]: this is true for both human and ecological risks assessments.

For the protection of aquatic ecosystems, Seifert [78] suggested an interim trigger level of 100 μgL^−1^ total CYN (*i.e.*, extracellular plus intracellular quantities) was an appropriate value. This was based on ecotoxicity work showing that sub-lethal and lethal toxicities are rarely significant below this level. Kinnear *et al.* [90] instead proposed a threshold system based on a combination of factors including toxin concentration, the proportion of toxin present in the intracellular form, and the total cell number. In agreement with Seifert, toxin concentrations exceeding 100 μg L^−1^ were assessed as being of particularly high risk (Kinnear *et al.* 2009). However, the emerging research indicates that bioaccumulation of CYN can occur even at trace quantities of exposure to the toxin: this suggests that caution must be exercised, particularly when developing risk assessment guidelines for aquatic ecosystem health. Furthermore, such guidelines need to be proactive, rather than reactive, if they are to remove or reduce the risks of bioaccumulation during toxin-producing blooms.

## 4. Where to Next?

As climate change and other pressure increases the range of CYN producers into subtropical and temperate climes, more animals and plants will become vulnerable to CYN bioaccumulation and biomagnification. In turn, this will lead to accompanying human and ecosystem health implications. Unfortunately, toxicological research is often prioritised based only on its ability to inform human health risk assessments. Where bioaccumulation studies have been pursued, this is done largely from the perspective of food web toxin transfer and hence the potential for human consumption. For example, the review of Ibelings and Chorus [12] well summarised the status of research on accumulation of cyanotoxins, but only from the perspective of public health outcomes following seafood consumption (fish, crayfish, prawns and mussels). Smith *et al.* [127] also provided a reviewed focused on implications for aquaculture systems.

The near-term research priorities for CYN identified by Pegram *et al.* [87] and others in that volume included studies of genotoxicity, carcinogenicity and toxicokinetics, as well as immunologic, reproductive, and developmental effects, and better descriptions of CYN activation, distribution and binding. With the exception of trials examining distribution and binding, few of these seem directly relevant to understanding CYN’s bioaccumulative potential. Studies to clarify the mechanism of CYN toxicity, in both *in vitro* and *in vivo* models, will be useful in linking with bioaccumulation work. Studies on simultaneous and sequential exposure to toxins, and to toxin mixtures, should be done to explore the possibility for additive, synergistic or antagonistic effects. This is vitally important since single cylindrospermopsins almost never occur in nature [87].

Despite the advances in toxin detection in water, the problem of detecting toxins that become bound into tissues remains unresolved. Also, in terms of laboratory studies, recovery and purification of CYN from spent culture media remains the most effective way to obtain quality toxin with which to work [78]. Commercial standards for cylindrospermopsin are slowly becoming available, as are ELISA-based detection kits [87]. However, few laboratories are suppliers, so the further development of both these resources continues to be of high priority. Radio-labelling of CYN would enable studies on whether the toxin can permeate all cell membranes [87]. However, only one report has been made of such a study using ^14^C-labelling in mice [97].

In bioaccumulation studies, there is a need to emphasize the use of environmentally realistic test concentrations in laboratory applications. The highest published field concentration of CYN from *C. raciborskii* is currently 589 ug L^−1^ [91] although an earlier study found 1.5 mg L^−1^ [128]. Sivonen & Jones [18] also recorded CYN up to 5,500 μg g^−1^, but this was from a sample of unidentified dried bloom material. Nevertheless, subtropical Australian environments more typically experience concentrations less than 20 μg L^−1^ [25]. By comparison, cultured *C. raciborskii* populations can be much more productive at up to 2.5 mg L^−1^ [129]. Thus, studies hoping to examine bioaccumulation potential should reflect this range of concentrations, as distinct from ecotoxicity testing where much high nominal CYN concentrations have been used (e.g., 5–1,000 μg mL^−1^ in Metcalf *et al.* [81]). Field studies often represent the ‘pinnacle’ of environmentally-relevant of research, but the spatial and temporal variation that is expected in CYN production, and in *C. raciborskii* and other blooms, makes it a difficult task to accurately determine what exposure levels are.

## 5. Conclusions

Serious human health and ecological effects are posed by algal blooms containing CYN. The distribution of CYN producers is growing; conditions predisposing plant and animals to bioaccumulation are more numerous than ever before. To date, the research focus for CYN has been squarely on human health risks, with few studies on environmental effects, and fewer still on bioaccumulation. Data showing the ability of CYN to accumulate has been available since 1999, but compared with toxicological work, few studies have been conducted on bioaccumulation overall. This is unfortunate given that CYN is growing in importance and bioaccumulation has important implications for human and ecological health risks. Without further studies, current risk assessments are almost certainly underestimating the overall risks of toxin-containing blooms.
